# Supplementary material for: An assessment model of bio-efficiency for container terminals in the presence of air emissions
Source: PLoS One. 2025 Mar 19;20(3):e0319423. doi: 10.1371/journal.pone.0319423 (PMC11957765; doi:10.1371/journal.pone.0319423)
Supplement: S1 Data — (DOCX) [file pone.0319423.s001.docx]

| DMUs | Employees | Container yard (m2) | STS cranes | Yard cranes | Air emissions (kg/TEU) | Container throughput (TEUs) |
| --- | --- | --- | --- | --- | --- | --- |
| CT.1 | 208 | 362,753 | 17 | 44 | 97.8 | 1,124,122 |
| CT.2 | 115 | 136,942 | 10 | 16 | 54.7 | 266,583 |
| CT.3 | 78 | 151,131 | 8 | 21 | 45.7 | 714,838 |
| CT.4 | 143 | 230,261 | 10 | 19 | 56.3 | 513,295 |
| CT.5 | 238 | 902,329 | 22 | 31 | 76.8 | 1,180,129 |
| CT.6 | 175 | 351,182 | 9 | 39 | 59.4 | 196,132 |
| CT.7 | 83 | 92,972 | 7 | 13 | 40.2 | 112,135 |
| CT.8 | 64 | 232,133 | 13 | 24 | 51.2 | 128,910 |
| CT.9 | 47 | 149,045 | 10 | 11 | 83.8 | 152,441 |
| CT.10 | 207 | 451,358 | 23 | 36 | 93.3 | 1,090,609 |
| CT.11 | 89 | 127,310 | 12 | 21 | 56.2 | 134,102 |
| CT.12 | 153 | 331,280 | 11 | 25 | 80.4 | 410,913 |

Bootstrap results:

| CT.1 | CT.2 | CT.3 | CT.4 | CT.5 | CT.6 | CT.7 | CT.8 | CT.9 | CT.10 | CT.11 | CT.12 |
| --- | --- | --- | --- | --- | --- | --- | --- | --- | --- | --- | --- |
| 0.652 | 0.246 | 0.910 | 0.480 | 0.900 | 0.111 | 0.148 | 0.413 | 0.213 | 0.606 | 0.125 | 0.248 |
| 0.598 | 0.248 | 0.990 | 0.420 | 0.900 | 0.113 | 0.133 | 0.117 | 0.199 | 0.517 | 0.133 | 0.245 |
| 0.646 | 0.294 | 0.920 | 0.471 | 0.940 | 0.421 | 0.161 | 0.113 | 0.179 | 0.523 | 0.113 | 0.299 |
| 0.592 | 0.292 | 0.940 | 0.448 | 0.940 | 0.120 | 0.135 | 0.112 | 0.199 | 0.606 | 0.133 | 0.299 |
| 0.562 | 0.278 | 0.940 | 0.444 | 0.940 | 0.122 | 0.149 | 0.109 | 0.189 | 0.612 | 0.130 | 0.299 |
| 0.568 | 0.259 | 0.940 | 0.425 | 0.940 | 0.114 | 0.155 | 0.108 | 0.203 | 0.539 | 0.127 | 0.280 |
| 0.568 | 0.286 | 0.980 | 0.434 | 0.940 | 0.120 | 0.163 | 0.103 | 0.193 | 0.567 | 0.118 | 0.275 |
| 0.568 | 0.251 | 0.980 | 0.476 | 0.940 | 0.123 | 0.144 | 0.098 | 0.181 | 0.550 | 0.112 | 0.248 |
| 0.580 | 0.294 | 0.990 | 0.453 | 0.940 | 0.108 | 0.160 | 0.107 | 0.203 | 0.578 | 0.113 | 0.261 |
| 0.586 | 0.243 | 1.000 | 0.476 | 0.940 | 0.106 | 0.160 | 0.113 | 0.213 | 0.500 | 0.118 | 0.250 |
| 0.640 | 0.265 | 1.000 | 0.467 | 0.940 | 0.111 | 0.141 | 0.105 | 0.181 | 0.562 | 0.128 | 0.299 |
| 0.634 | 0.265 | 1.000 | 0.462 | 0.940 | 0.119 | 0.147 | 0.096 | 0.211 | 0.600 | 0.119 | 0.253 |
| 0.550 | 0.284 | 1.000 | 0.494 | 0.900 | 0.111 | 0.152 | 0.106 | 0.205 | 0.556 | 0.121 | 0.256 |
| 0.634 | 0.275 | 1.000 | 0.499 | 0.940 | 0.110 | 0.152 | 0.105 | 0.205 | 0.567 | 0.113 | 0.258 |
| 0.568 | 0.297 | 1.000 | 0.471 | 0.940 | 0.120 | 0.148 | 0.098 | 0.215 | 0.512 | 0.129 | 0.283 |
| 0.544 | 0.251 | 1.000 | 0.508 | 0.940 | 0.103 | 0.147 | 0.116 | 0.197 | 0.500 | 0.134 | 0.245 |
| 0.574 | 0.246 | 1.000 | 0.508 | 0.940 | 0.115 | 0.152 | 0.098 | 0.215 | 0.545 | 0.122 | 0.264 |
| 0.562 | 0.284 | 1.000 | 0.453 | 0.940 | 0.112 | 0.145 | 0.101 | 0.203 | 0.578 | 0.116 | 0.248 |
| 0.616 | 0.278 | 1.000 | 0.476 | 0.940 | 0.104 | 0.135 | 0.114 | 0.191 | 0.506 | 0.134 | 0.253 |
| 0.604 | 0.289 | 1.000 | 0.420 | 0.940 | 0.115 | 0.149 | 0.108 | 0.191 | 0.612 | 0.118 | 0.256 |
| 0.610 | 0.262 | 1.000 | 0.490 | 0.940 | 0.124 | 0.139 | 0.117 | 0.197 | 0.523 | 0.117 | 0.277 |
| 0.562 | 0.265 | 1.000 | 0.467 | 0.940 | 0.106 | 0.136 | 0.114 | 0.199 | 0.534 | 0.119 | 0.250 |
| 0.710 | 0.281 | 1.000 | 0.430 | 0.940 | 0.115 | 0.149 | 0.109 | 0.187 | 0.562 | 0.129 | 0.288 |
| 0.544 | 0.246 | 1.000 | 0.457 | 0.900 | 0.106 | 0.135 | 0.110 | 0.181 | 0.600 | 0.112 | 0.272 |
| 0.746 | 0.275 | 1.000 | 0.462 | 0.940 | 0.121 | 0.145 | 0.102 | 0.179 | 0.550 | 0.129 | 0.267 |
| 0.550 | 0.281 | 1.000 | 0.416 | 0.940 | 0.111 | 0.145 | 0.108 | 0.195 | 0.500 | 0.135 | 0.294 |
| 0.652 | 0.243 | 1.000 | 0.490 | 0.940 | 0.106 | 0.144 | 0.110 | 0.209 | 0.562 | 0.134 | 0.258 |
| 0.664 | 0.243 | 1.000 | 0.420 | 0.940 | 0.107 | 0.161 | 0.117 | 0.211 | 0.606 | 0.116 | 0.288 |
| 0.616 | 0.278 | 0.900 | 0.471 | 0.950 | 0.111 | 0.154 | 0.109 | 0.201 | 0.573 | 0.128 | 0.291 |
| 0.586 | 0.267 | 0.910 | 0.434 | 0.950 | 0.113 | 0.145 | 0.118 | 0.207 | 0.612 | 0.113 | 0.286 |
| 0.556 | 0.246 | 0.920 | 0.467 | 0.950 | 0.107 | 0.144 | 0.105 | 0.189 | 0.589 | 0.114 | 0.277 |
| 0.652 | 0.267 | 0.920 | 0.490 | 0.950 | 0.113 | 0.149 | 0.102 | 0.183 | 0.606 | 0.123 | 0.258 |
| 0.562 | 0.270 | 0.920 | 0.420 | 0.950 | 0.115 | 0.139 | 0.102 | 0.201 | 0.506 | 0.114 | 0.258 |
| 0.598 | 0.243 | 0.940 | 0.416 | 0.950 | 0.112 | 0.151 | 0.116 | 0.195 | 0.506 | 0.121 | 0.275 |
| 0.634 | 0.289 | 1.000 | 0.467 | 0.900 | 0.103 | 0.161 | 0.516 | 0.185 | 0.517 | 0.125 | 0.299 |
| 0.658 | 0.257 | 0.960 | 0.420 | 0.950 | 0.113 | 0.163 | 0.102 | 0.179 | 0.595 | 0.112 | 0.269 |
| 0.592 | 0.281 | 0.960 | 0.434 | 0.950 | 0.107 | 0.149 | 0.098 | 0.205 | 0.612 | 0.112 | 0.294 |
| 0.574 | 0.294 | 0.960 | 0.494 | 0.950 | 0.122 | 0.152 | 0.117 | 0.217 | 0.600 | 0.123 | 0.296 |
| 0.616 | 0.292 | 0.990 | 0.467 | 0.950 | 0.107 | 0.163 | 0.108 | 0.201 | 0.578 | 0.133 | 0.258 |
| 0.592 | 0.265 | 0.990 | 0.444 | 0.950 | 0.110 | 0.155 | 0.108 | 0.181 | 0.584 | 0.133 | 0.291 |
| 0.664 | 0.273 | 1.000 | 0.462 | 0.950 | 0.124 | 0.133 | 0.101 | 0.201 | 0.534 | 0.125 | 0.256 |
| 0.616 | 0.273 | 1.000 | 0.471 | 0.950 | 0.108 | 0.147 | 0.097 | 0.187 | 0.556 | 0.127 | 0.283 |
| 0.592 | 0.278 | 1.000 | 0.471 | 0.950 | 0.105 | 0.157 | 0.116 | 0.197 | 0.512 | 0.132 | 0.286 |
| 0.598 | 0.292 | 1.000 | 0.471 | 0.950 | 0.111 | 0.133 | 0.098 | 0.215 | 0.523 | 0.112 | 0.272 |
| 0.646 | 0.248 | 1.000 | 0.504 | 0.950 | 0.118 | 0.160 | 0.109 | 0.191 | 0.578 | 0.114 | 0.264 |
| 0.628 | 0.257 | 1.000 | 0.453 | 0.900 | 0.103 | 0.155 | 0.103 | 0.191 | 0.600 | 0.134 | 0.258 |
| 0.634 | 0.275 | 1.000 | 0.490 | 0.950 | 0.115 | 0.161 | 0.108 | 0.189 | 0.506 | 0.114 | 0.250 |
| 0.664 | 0.248 | 0.900 | 0.425 | 0.960 | 0.105 | 0.145 | 0.107 | 0.185 | 0.584 | 0.116 | 0.291 |
| 0.604 | 0.251 | 0.900 | 0.448 | 0.960 | 0.120 | 0.151 | 0.118 | 0.207 | 0.556 | 0.114 | 0.248 |
| 0.640 | 0.278 | 0.900 | 0.430 | 0.960 | 0.115 | 0.157 | 0.100 | 0.185 | 0.589 | 0.125 | 0.258 |
| 0.580 | 0.297 | 0.900 | 0.508 | 0.960 | 0.106 | 0.133 | 0.101 | 0.193 | 0.512 | 0.135 | 0.253 |
| 0.574 | 0.292 | 0.910 | 0.471 | 0.960 | 0.116 | 0.139 | 0.102 | 0.193 | 0.562 | 0.129 | 0.264 |
| 0.544 | 0.292 | 0.920 | 0.444 | 0.960 | 0.122 | 0.149 | 0.101 | 0.217 | 0.612 | 0.122 | 0.248 |
| 0.550 | 0.267 | 0.930 | 0.453 | 0.960 | 0.103 | 0.151 | 0.109 | 0.197 | 0.517 | 0.134 | 0.296 |
| 0.628 | 0.297 | 0.950 | 0.439 | 0.960 | 0.122 | 0.147 | 0.105 | 0.219 | 0.606 | 0.134 | 0.291 |
| 0.562 | 0.286 | 0.960 | 0.439 | 0.960 | 0.116 | 0.158 | 0.106 | 0.181 | 0.573 | 0.111 | 0.245 |
| 0.580 | 0.267 | 1.000 | 0.476 | 0.900 | 0.124 | 0.145 | 0.111 | 0.213 | 0.595 | 0.413 | 0.250 |
| 0.640 | 0.248 | 0.980 | 0.434 | 0.960 | 0.114 | 0.138 | 0.111 | 0.217 | 0.506 | 0.121 | 0.283 |
| 0.544 | 0.273 | 0.990 | 0.425 | 0.960 | 0.123 | 0.154 | 0.109 | 0.201 | 0.567 | 0.132 | 0.286 |
| 0.664 | 0.286 | 0.990 | 0.416 | 0.960 | 0.116 | 0.135 | 0.109 | 0.211 | 0.506 | 0.122 | 0.267 |
| 0.592 | 0.254 | 1.000 | 0.494 | 0.960 | 0.104 | 0.142 | 0.114 | 0.197 | 0.506 | 0.116 | 0.280 |
| 0.574 | 0.259 | 1.000 | 0.457 | 0.960 | 0.103 | 0.157 | 0.113 | 0.195 | 0.578 | 0.113 | 0.261 |
| 0.622 | 0.275 | 1.000 | 0.467 | 0.960 | 0.114 | 0.142 | 0.105 | 0.187 | 0.500 | 0.124 | 0.250 |
| 0.658 | 0.259 | 1.000 | 0.453 | 0.960 | 0.111 | 0.139 | 0.106 | 0.181 | 0.539 | 0.118 | 0.256 |
| 0.556 | 0.294 | 1.000 | 0.476 | 0.960 | 0.107 | 0.151 | 0.111 | 0.217 | 0.556 | 0.112 | 0.256 |
| 0.664 | 0.297 | 1.000 | 0.448 | 0.960 | 0.121 | 0.145 | 0.097 | 0.203 | 0.556 | 0.124 | 0.269 |
| 0.616 | 0.254 | 1.000 | 0.499 | 0.960 | 0.113 | 0.148 | 0.108 | 0.179 | 0.606 | 0.118 | 0.286 |
| 0.556 | 0.254 | 1.000 | 0.457 | 0.900 | 0.114 | 0.154 | 0.105 | 0.203 | 0.612 | 0.127 | 0.248 |
| 0.622 | 0.248 | 1.000 | 0.430 | 0.960 | 0.120 | 0.148 | 0.103 | 0.211 | 0.550 | 0.127 | 0.272 |
| 0.610 | 0.259 | 1.000 | 0.485 | 0.960 | 0.110 | 0.152 | 0.118 | 0.205 | 0.567 | 0.118 | 0.269 |
| 0.574 | 0.275 | 1.000 | 0.467 | 0.960 | 0.114 | 0.158 | 0.118 | 0.189 | 0.600 | 0.119 | 0.286 |
| 0.652 | 0.265 | 1.000 | 0.508 | 0.960 | 0.116 | 0.148 | 0.118 | 0.199 | 0.606 | 0.135 | 0.283 |
| 0.580 | 0.254 | 1.000 | 0.494 | 0.960 | 0.113 | 0.144 | 0.109 | 0.215 | 0.500 | 0.125 | 0.283 |
| 0.562 | 0.270 | 1.000 | 0.439 | 0.960 | 0.119 | 0.145 | 0.098 | 0.203 | 0.589 | 0.130 | 0.275 |
| 0.598 | 0.259 | 1.000 | 0.490 | 0.960 | 0.113 | 0.163 | 0.103 | 0.183 | 0.539 | 0.129 | 0.261 |
| 0.580 | 0.246 | 1.000 | 0.504 | 0.960 | 0.124 | 0.142 | 0.109 | 0.201 | 0.500 | 0.133 | 0.275 |
| 0.556 | 0.248 | 0.900 | 0.444 | 0.970 | 0.506 | 0.161 | 0.105 | 0.205 | 0.573 | 0.134 | 0.256 |
| 0.658 | 0.273 | 0.900 | 0.499 | 0.970 | 0.105 | 0.144 | 0.109 | 0.207 | 0.517 | 0.130 | 0.258 |
| 0.556 | 0.254 | 1.000 | 0.444 | 0.900 | 0.115 | 0.141 | 0.400 | 0.187 | 0.595 | 0.117 | 0.248 |
| 0.646 | 0.278 | 0.900 | 0.453 | 0.970 | 0.116 | 0.135 | 0.106 | 0.181 | 0.567 | 0.134 | 0.275 |
| 0.610 | 0.292 | 0.900 | 0.444 | 0.970 | 0.118 | 0.138 | 0.111 | 0.195 | 0.600 | 0.118 | 0.264 |
| 0.622 | 0.270 | 0.900 | 0.457 | 0.970 | 0.108 | 0.151 | 0.108 | 0.191 | 0.512 | 0.122 | 0.299 |
| 0.622 | 0.248 | 0.900 | 0.434 | 0.970 | 0.108 | 0.154 | 0.118 | 0.201 | 0.545 | 0.113 | 0.250 |
| 0.574 | 0.251 | 0.910 | 0.508 | 0.970 | 0.122 | 0.145 | 0.107 | 0.211 | 0.523 | 0.129 | 0.277 |
| 0.562 | 0.262 | 0.940 | 0.471 | 0.970 | 0.106 | 0.147 | 0.116 | 0.191 | 0.517 | 0.123 | 0.288 |
| 0.562 | 0.251 | 0.950 | 0.430 | 0.970 | 0.107 | 0.145 | 0.096 | 0.201 | 0.562 | 0.133 | 0.261 |
| 0.664 | 0.246 | 0.950 | 0.453 | 0.970 | 0.114 | 0.133 | 0.112 | 0.207 | 0.567 | 0.134 | 0.250 |
| 0.628 | 0.243 | 0.950 | 0.499 | 0.970 | 0.120 | 0.148 | 0.109 | 0.219 | 0.500 | 0.130 | 0.261 |
| 0.622 | 0.278 | 0.960 | 0.467 | 0.970 | 0.105 | 0.163 | 0.111 | 0.211 | 0.506 | 0.111 | 0.277 |
| 0.556 | 0.259 | 1.000 | 0.448 | 0.900 | 0.121 | 0.147 | 0.118 | 0.199 | 0.589 | 0.116 | 0.272 |
| 0.604 | 0.294 | 0.970 | 0.471 | 0.970 | 0.105 | 0.148 | 0.098 | 0.211 | 0.500 | 0.133 | 0.299 |
| 0.556 | 0.281 | 0.970 | 0.476 | 0.970 | 0.123 | 0.144 | 0.096 | 0.207 | 0.600 | 0.133 | 0.267 |
| 0.634 | 0.294 | 0.980 | 0.434 | 0.970 | 0.112 | 0.133 | 0.102 | 0.185 | 0.556 | 0.111 | 0.267 |
| 0.616 | 0.246 | 0.990 | 0.499 | 0.970 | 0.114 | 0.136 | 0.109 | 0.193 | 0.512 | 0.122 | 0.299 |
| 0.556 | 0.292 | 1.000 | 0.480 | 0.970 | 0.120 | 0.136 | 0.105 | 0.203 | 0.562 | 0.129 | 0.288 |
| 0.544 | 0.265 | 1.000 | 0.416 | 0.970 | 0.112 | 0.144 | 0.109 | 0.207 | 0.523 | 0.118 | 0.277 |
| 0.616 | 0.248 | 1.000 | 0.485 | 0.970 | 0.104 | 0.138 | 0.117 | 0.209 | 0.595 | 0.113 | 0.294 |
| 0.574 | 0.257 | 1.000 | 0.439 | 0.970 | 0.118 | 0.152 | 0.105 | 0.191 | 0.578 | 0.117 | 0.286 |
| 0.580 | 0.289 | 1.000 | 0.499 | 0.970 | 0.121 | 0.147 | 0.117 | 0.183 | 0.573 | 0.121 | 0.296 |
| 0.586 | 0.289 | 1.000 | 0.471 | 0.970 | 0.120 | 0.154 | 0.101 | 0.209 | 0.512 | 0.118 | 0.286 |
| 0.598 | 0.286 | 1.000 | 0.485 | 0.900 | 0.105 | 0.161 | 0.110 | 0.179 | 0.556 | 0.134 | 0.299 |
| 0.592 | 0.259 | 1.000 | 0.420 | 0.970 | 0.123 | 0.154 | 0.109 | 0.219 | 0.556 | 0.129 | 0.277 |
| 0.562 | 0.251 | 1.000 | 0.480 | 0.970 | 0.119 | 0.151 | 0.118 | 0.179 | 0.562 | 0.125 | 0.291 |
| 0.544 | 0.294 | 1.000 | 0.508 | 0.970 | 0.118 | 0.144 | 0.103 | 0.195 | 0.550 | 0.123 | 0.245 |
| 0.634 | 0.297 | 1.000 | 0.425 | 0.970 | 0.119 | 0.163 | 0.107 | 0.211 | 0.606 | 0.118 | 0.269 |
| 0.622 | 0.278 | 1.000 | 0.430 | 0.970 | 0.105 | 0.160 | 0.117 | 0.183 | 0.584 | 0.124 | 0.258 |
| 0.586 | 0.248 | 1.000 | 0.457 | 0.970 | 0.108 | 0.161 | 0.118 | 0.185 | 0.545 | 0.113 | 0.248 |
| 0.610 | 0.248 | 1.000 | 0.508 | 0.970 | 0.111 | 0.138 | 0.112 | 0.199 | 0.556 | 0.118 | 0.299 |
| 0.640 | 0.275 | 1.000 | 0.420 | 0.970 | 0.123 | 0.160 | 0.116 | 0.187 | 0.606 | 0.111 | 0.283 |
| 0.640 | 0.267 | 1.000 | 0.457 | 0.970 | 0.103 | 0.135 | 0.107 | 0.197 | 0.612 | 0.129 | 0.253 |
| 0.592 | 0.246 | 1.000 | 0.494 | 0.970 | 0.119 | 0.163 | 0.103 | 0.207 | 0.517 | 0.124 | 0.250 |
| 0.568 | 0.284 | 0.910 | 0.439 | 0.900 | 0.104 | 0.163 | 0.114 | 0.207 | 0.528 | 0.124 | 0.272 |
| 0.544 | 0.251 | 1.000 | 0.416 | 0.900 | 0.119 | 0.161 | 0.104 | 0.179 | 0.539 | 0.128 | 0.261 |
| 0.586 | 0.254 | 1.000 | 0.471 | 0.970 | 0.123 | 0.148 | 0.113 | 0.197 | 0.534 | 0.112 | 0.245 |
| 0.640 | 0.284 | 1.000 | 0.480 | 0.970 | 0.106 | 0.163 | 0.113 | 0.211 | 0.539 | 0.132 | 0.291 |
| 0.574 | 0.284 | 0.900 | 0.508 | 0.980 | 0.105 | 0.145 | 0.112 | 0.217 | 0.612 | 0.112 | 0.261 |
| 0.610 | 0.248 | 0.900 | 0.457 | 0.980 | 0.115 | 0.149 | 0.098 | 0.201 | 0.595 | 0.118 | 0.277 |
| 0.574 | 0.267 | 0.900 | 0.420 | 0.980 | 0.122 | 0.161 | 0.114 | 0.215 | 0.545 | 0.125 | 0.283 |
| 0.658 | 0.284 | 0.910 | 0.471 | 0.980 | 0.121 | 0.135 | 0.113 | 0.215 | 0.550 | 0.123 | 0.264 |
| 0.592 | 0.297 | 0.910 | 0.485 | 0.980 | 0.120 | 0.151 | 0.117 | 0.219 | 0.528 | 0.127 | 0.245 |
| 0.574 | 0.292 | 0.920 | 0.448 | 0.980 | 0.119 | 0.149 | 0.102 | 0.183 | 0.556 | 0.132 | 0.264 |
| 0.604 | 0.286 | 0.930 | 0.457 | 0.980 | 0.111 | 0.149 | 0.101 | 0.203 | 0.539 | 0.127 | 0.294 |
| 0.604 | 0.275 | 0.930 | 0.425 | 0.980 | 0.105 | 0.141 | 0.114 | 0.191 | 0.534 | 0.129 | 0.283 |
| 0.586 | 0.275 | 1.000 | 0.430 | 0.900 | 0.110 | 0.160 | 0.118 | 0.207 | 0.500 | 0.119 | 0.272 |
| 0.592 | 0.243 | 0.930 | 0.485 | 0.980 | 0.116 | 0.138 | 0.117 | 0.203 | 0.523 | 0.133 | 0.272 |
| 0.616 | 0.294 | 0.930 | 0.485 | 0.980 | 0.119 | 0.139 | 0.101 | 0.219 | 0.600 | 0.125 | 0.277 |
| 0.550 | 0.292 | 0.940 | 0.448 | 0.980 | 0.115 | 0.151 | 0.103 | 0.213 | 0.534 | 0.134 | 0.256 |
| 0.598 | 0.270 | 0.940 | 0.467 | 0.980 | 0.110 | 0.148 | 0.118 | 0.189 | 0.556 | 0.117 | 0.272 |
| 0.610 | 0.275 | 0.950 | 0.499 | 0.980 | 0.102 | 0.149 | 0.118 | 0.181 | 0.506 | 0.129 | 0.256 |
| 0.652 | 0.267 | 0.980 | 0.444 | 0.980 | 0.106 | 0.145 | 0.111 | 0.211 | 0.589 | 0.121 | 0.299 |
| 0.544 | 0.270 | 0.980 | 0.485 | 0.980 | 0.118 | 0.142 | 0.111 | 0.179 | 0.534 | 0.125 | 0.283 |
| 0.646 | 0.281 | 0.990 | 0.453 | 0.980 | 0.113 | 0.163 | 0.117 | 0.183 | 0.556 | 0.118 | 0.294 |
| 0.652 | 0.294 | 0.990 | 0.453 | 0.980 | 0.108 | 0.151 | 0.096 | 0.179 | 0.606 | 0.128 | 0.272 |
| 0.544 | 0.248 | 1.000 | 0.453 | 0.980 | 0.422 | 0.154 | 0.118 | 0.183 | 0.517 | 0.112 | 0.294 |
| 0.646 | 0.243 | 0.900 | 0.476 | 0.910 | 0.102 | 0.158 | 0.110 | 0.187 | 0.567 | 0.132 | 0.267 |
| 0.598 | 0.267 | 1.000 | 0.416 | 0.980 | 0.115 | 0.157 | 0.113 | 0.183 | 0.595 | 0.127 | 0.261 |
| 0.658 | 0.294 | 1.000 | 0.453 | 0.980 | 0.107 | 0.135 | 0.109 | 0.207 | 0.562 | 0.135 | 0.245 |
| 0.556 | 0.267 | 1.000 | 0.439 | 0.980 | 0.123 | 0.147 | 0.101 | 0.191 | 0.506 | 0.116 | 0.291 |
| 0.574 | 0.297 | 1.000 | 0.467 | 0.980 | 0.110 | 0.145 | 0.101 | 0.217 | 0.595 | 0.123 | 0.256 |
| 0.658 | 0.265 | 1.000 | 0.485 | 0.980 | 0.103 | 0.139 | 0.105 | 0.215 | 0.567 | 0.113 | 0.269 |
| 0.646 | 0.262 | 1.000 | 0.434 | 0.980 | 0.118 | 0.144 | 0.113 | 0.185 | 0.528 | 0.111 | 0.261 |
| 0.604 | 0.294 | 1.000 | 0.453 | 0.980 | 0.122 | 0.145 | 0.114 | 0.213 | 0.523 | 0.125 | 0.286 |
| 0.574 | 0.281 | 0.900 | 0.480 | 0.990 | 0.123 | 0.149 | 0.112 | 0.181 | 0.567 | 0.112 | 0.294 |
| 0.616 | 0.294 | 0.900 | 0.476 | 0.990 | 0.108 | 0.163 | 0.106 | 0.179 | 0.606 | 0.135 | 0.261 |
| 0.592 | 0.248 | 0.910 | 0.425 | 0.990 | 0.111 | 0.154 | 0.096 | 0.215 | 0.534 | 0.116 | 0.258 |
| 0.622 | 0.259 | 0.900 | 0.462 | 0.910 | 0.106 | 0.138 | 0.102 | 0.181 | 0.606 | 0.116 | 0.253 |
| 0.646 | 0.267 | 0.920 | 0.425 | 0.990 | 0.106 | 0.163 | 0.104 | 0.181 | 0.528 | 0.121 | 0.288 |
| 0.568 | 0.248 | 0.920 | 0.434 | 0.990 | 0.122 | 0.152 | 0.106 | 0.187 | 0.528 | 0.116 | 0.250 |
| 0.658 | 0.294 | 0.920 | 0.494 | 0.990 | 0.108 | 0.152 | 0.103 | 0.199 | 0.589 | 0.124 | 0.294 |
| 0.610 | 0.251 | 0.930 | 0.462 | 0.990 | 0.106 | 0.148 | 0.102 | 0.185 | 0.528 | 0.130 | 0.264 |
| 0.640 | 0.259 | 0.930 | 0.439 | 0.990 | 0.107 | 0.152 | 0.103 | 0.185 | 0.584 | 0.130 | 0.256 |
| 0.616 | 0.273 | 0.960 | 0.499 | 0.990 | 0.119 | 0.158 | 0.102 | 0.187 | 0.573 | 0.123 | 0.277 |
| 0.556 | 0.281 | 0.970 | 0.420 | 0.990 | 0.114 | 0.148 | 0.118 | 0.185 | 0.578 | 0.121 | 0.272 |
| 0.556 | 0.294 | 0.980 | 0.508 | 0.990 | 0.102 | 0.141 | 0.096 | 0.201 | 0.534 | 0.123 | 0.264 |
| 0.592 | 0.259 | 0.990 | 0.462 | 0.990 | 0.115 | 0.135 | 0.098 | 0.183 | 0.556 | 0.133 | 0.280 |
| 0.634 | 1.000 | 1.000 | 0.471 | 0.990 | 0.205 | 0.135 | 0.110 | 0.217 | 0.600 | 0.111 | 0.248 |
| 0.616 | 0.262 | 0.910 | 0.485 | 0.910 | 0.123 | 0.152 | 0.596 | 0.211 | 0.506 | 0.413 | 0.269 |
| 0.610 | 0.257 | 1.000 | 0.457 | 0.990 | 0.113 | 0.136 | 0.096 | 0.181 | 0.523 | 0.125 | 0.291 |
| 0.616 | 0.262 | 1.000 | 0.430 | 0.990 | 0.124 | 0.141 | 0.098 | 0.201 | 0.584 | 0.127 | 0.291 |
| 0.562 | 0.270 | 1.000 | 0.434 | 0.990 | 0.115 | 0.158 | 0.111 | 0.219 | 0.550 | 0.125 | 0.248 |
| 0.556 | 0.278 | 1.000 | 0.476 | 0.990 | 0.114 | 0.133 | 0.113 | 0.201 | 0.534 | 0.117 | 0.275 |
| 0.646 | 0.259 | 1.000 | 0.471 | 0.990 | 0.113 | 0.157 | 0.108 | 0.207 | 0.556 | 0.122 | 0.294 |
| 0.574 | 0.275 | 1.000 | 0.439 | 0.990 | 0.123 | 0.160 | 0.108 | 0.181 | 0.562 | 0.121 | 0.291 |
| 0.598 | 0.270 | 1.000 | 0.453 | 0.990 | 0.110 | 0.139 | 0.117 | 0.189 | 0.550 | 0.124 | 0.283 |
| 0.628 | 0.284 | 1.000 | 0.416 | 0.990 | 0.113 | 0.145 | 0.117 | 0.179 | 0.567 | 0.114 | 0.296 |
| 0.610 | 0.262 | 1.000 | 0.467 | 0.990 | 0.116 | 0.163 | 0.113 | 0.217 | 0.550 | 0.128 | 0.296 |
| 0.664 | 0.251 | 1.000 | 0.504 | 0.990 | 0.115 | 0.154 | 0.111 | 0.217 | 0.550 | 0.122 | 0.283 |
| 0.592 | 0.289 | 0.920 | 0.480 | 0.910 | 0.116 | 0.142 | 0.108 | 0.207 | 0.528 | 0.134 | 0.272 |
| 0.628 | 0.257 | 1.000 | 0.490 | 0.990 | 0.123 | 0.144 | 0.112 | 0.203 | 0.556 | 0.111 | 0.299 |
| 0.586 | 0.254 | 1.000 | 0.504 | 0.990 | 0.104 | 0.163 | 0.110 | 0.183 | 0.600 | 0.118 | 0.250 |
| 0.652 | 0.262 | 1.000 | 0.494 | 0.990 | 0.124 | 0.145 | 0.100 | 0.187 | 0.534 | 0.125 | 0.294 |
| 0.550 | 0.262 | 1.000 | 0.471 | 0.990 | 0.122 | 0.138 | 0.100 | 0.205 | 0.523 | 0.116 | 0.280 |
| 0.550 | 0.267 | 1.000 | 0.453 | 0.990 | 0.113 | 0.144 | 0.113 | 0.201 | 0.500 | 0.127 | 0.280 |
| 0.640 | 0.281 | 0.900 | 0.480 | 1.000 | 0.106 | 0.154 | 0.097 | 0.203 | 0.506 | 0.111 | 0.280 |
| 0.652 | 0.262 | 0.900 | 0.434 | 1.000 | 0.106 | 0.160 | 0.108 | 0.203 | 0.600 | 0.123 | 0.245 |
| 0.652 | 0.273 | 0.920 | 0.453 | 1.000 | 0.102 | 0.147 | 0.112 | 0.207 | 0.506 | 0.123 | 0.261 |
| 0.664 | 0.248 | 0.920 | 0.499 | 1.000 | 0.115 | 0.158 | 0.106 | 0.205 | 0.573 | 0.112 | 0.256 |
| 0.592 | 0.251 | 0.930 | 0.444 | 1.000 | 0.106 | 0.149 | 0.102 | 0.205 | 0.584 | 0.129 | 0.253 |
| 0.544 | 0.281 | 0.930 | 0.448 | 0.910 | 0.103 | 0.135 | 0.096 | 0.183 | 0.612 | 0.133 | 0.256 |
| 0.556 | 0.292 | 0.960 | 0.448 | 1.000 | 0.104 | 0.145 | 0.104 | 0.185 | 0.545 | 0.127 | 0.258 |
| 0.658 | 0.267 | 0.980 | 0.462 | 1.000 | 0.122 | 0.133 | 0.108 | 0.181 | 0.534 | 0.128 | 0.272 |
| 0.592 | 0.267 | 0.980 | 0.471 | 1.000 | 0.115 | 0.133 | 0.117 | 0.179 | 0.573 | 0.132 | 0.288 |
| 0.604 | 0.284 | 0.990 | 0.453 | 1.000 | 0.116 | 0.141 | 0.097 | 0.195 | 0.573 | 0.134 | 0.294 |
| 0.592 | 0.251 | 0.990 | 0.439 | 1.000 | 0.115 | 0.133 | 0.103 | 0.179 | 0.534 | 0.122 | 0.280 |
| 0.604 | 0.265 | 1.000 | 0.504 | 1.000 | 0.118 | 0.158 | 0.110 | 0.217 | 0.528 | 0.112 | 0.288 |
| 0.544 | 0.294 | 1.000 | 0.471 | 1.000 | 0.116 | 0.149 | 0.117 | 0.187 | 0.578 | 0.122 | 0.269 |
| 0.562 | 0.243 | 1.000 | 0.494 | 1.000 | 0.115 | 0.142 | 0.118 | 0.189 | 0.612 | 0.134 | 0.291 |
| 0.622 | 0.289 | 1.000 | 0.439 | 1.000 | 0.118 | 0.161 | 0.112 | 0.201 | 0.534 | 0.119 | 0.269 |
| 0.550 | 0.262 | 1.000 | 0.430 | 1.000 | 0.113 | 0.155 | 0.117 | 0.197 | 0.523 | 0.122 | 0.269 |
| 0.562 | 0.275 | 0.930 | 0.416 | 0.910 | 0.113 | 0.161 | 0.113 | 0.179 | 0.512 | 0.124 | 0.261 |
| 0.598 | 0.243 | 1.000 | 0.480 | 1.000 | 0.124 | 0.135 | 0.117 | 0.219 | 0.506 | 0.133 | 0.253 |
| 0.640 | 0.270 | 1.000 | 0.480 | 1.000 | 0.119 | 0.152 | 0.102 | 0.205 | 0.512 | 0.121 | 0.272 |
| 0.604 | 0.262 | 1.000 | 0.453 | 1.000 | 0.114 | 0.149 | 0.102 | 0.201 | 0.512 | 0.127 | 0.256 |
| 0.586 | 0.275 | 1.000 | 0.485 | 1.000 | 0.114 | 0.161 | 0.101 | 0.185 | 0.550 | 0.116 | 0.299 |
| 0.592 | 0.267 | 1.000 | 0.457 | 1.000 | 0.124 | 0.155 | 0.113 | 0.205 | 0.545 | 0.130 | 0.248 |
| 0.574 | 0.254 | 1.000 | 0.434 | 1.000 | 0.103 | 0.161 | 0.100 | 0.203 | 0.556 | 0.113 | 0.272 |
| 0.586 | 0.281 | 1.000 | 0.444 | 1.000 | 0.123 | 0.144 | 0.100 | 0.185 | 0.550 | 0.130 | 0.253 |
| 0.610 | 0.273 | 1.000 | 0.490 | 1.000 | 0.103 | 0.149 | 0.108 | 0.201 | 0.539 | 0.111 | 0.277 |
| 0.628 | 0.284 | 1.000 | 0.416 | 1.000 | 0.108 | 0.163 | 0.116 | 0.191 | 0.567 | 0.117 | 0.256 |
| 0.610 | 0.262 | 1.000 | 0.457 | 1.000 | 0.103 | 0.148 | 0.105 | 0.183 | 0.550 | 0.116 | 0.248 |
| 0.664 | 0.259 | 0.940 | 0.471 | 0.910 | 0.116 | 0.136 | 0.111 | 0.183 | 0.573 | 0.133 | 0.299 |
| 0.646 | 0.248 | 1.000 | 0.425 | 1.000 | 0.110 | 0.154 | 0.103 | 0.181 | 0.589 | 0.119 | 0.286 |
| 0.604 | 0.246 | 1.000 | 0.499 | 1.000 | 0.118 | 0.149 | 0.105 | 0.207 | 0.523 | 0.133 | 0.277 |
| 0.610 | 0.265 | 0.910 | 0.476 | 1.000 | 0.103 | 0.163 | 0.096 | 0.211 | 0.573 | 0.127 | 0.283 |
| 0.616 | 0.289 | 0.910 | 0.430 | 1.000 | 0.108 | 0.163 | 0.117 | 0.197 | 0.534 | 0.121 | 0.250 |
| 0.640 | 0.270 | 0.920 | 0.485 | 1.000 | 0.103 | 0.145 | 0.116 | 0.185 | 0.517 | 0.113 | 0.291 |
| 0.568 | 0.297 | 0.940 | 0.485 | 1.000 | 0.122 | 0.145 | 0.109 | 0.197 | 0.534 | 0.124 | 0.264 |
| 0.646 | 0.246 | 0.940 | 0.425 | 1.000 | 0.103 | 0.138 | 0.101 | 0.203 | 0.500 | 0.114 | 0.286 |
| 0.574 | 0.275 | 0.950 | 0.439 | 1.000 | 0.119 | 0.148 | 0.107 | 0.197 | 0.517 | 0.112 | 0.294 |
| 0.562 | 0.292 | 0.990 | 0.434 | 1.000 | 0.121 | 0.161 | 0.111 | 0.219 | 0.567 | 0.129 | 0.250 |
| 0.616 | 0.248 | 1.000 | 0.416 | 1.000 | 0.124 | 0.138 | 0.104 | 0.199 | 0.562 | 0.114 | 0.250 |
| 0.556 | 0.284 | 0.950 | 0.457 | 0.910 | 0.113 | 0.142 | 0.096 | 0.215 | 0.567 | 0.121 | 0.253 |
| 0.586 | 0.281 | 1.000 | 0.425 | 1.000 | 0.103 | 0.139 | 0.106 | 0.215 | 0.517 | 0.118 | 0.288 |
| 0.604 | 0.273 | 1.000 | 0.453 | 1.000 | 0.124 | 0.133 | 0.117 | 0.201 | 0.584 | 0.123 | 0.261 |
| 0.556 | 0.289 | 1.000 | 0.416 | 1.000 | 0.112 | 0.154 | 0.109 | 0.217 | 0.573 | 0.123 | 0.267 |
| 0.610 | 0.297 | 1.000 | 0.494 | 1.000 | 0.123 | 0.163 | 0.108 | 0.185 | 0.595 | 0.129 | 0.272 |
| 0.592 | 0.273 | 1.000 | 0.476 | 1.000 | 0.112 | 0.155 | 0.100 | 0.185 | 0.500 | 0.123 | 0.286 |
| 0.604 | 0.273 | 1.000 | 0.425 | 1.000 | 0.106 | 0.154 | 0.104 | 0.201 | 0.562 | 0.129 | 0.291 |
| 0.622 | 0.243 | 0.910 | 0.476 | 1.000 | 0.120 | 0.152 | 0.116 | 0.215 | 0.584 | 0.122 | 0.250 |
| 0.592 | 0.259 | 0.910 | 0.499 | 1.000 | 0.110 | 0.133 | 0.103 | 0.187 | 0.562 | 0.118 | 0.283 |
| 0.622 | 0.292 | 0.920 | 0.485 | 1.000 | 0.103 | 0.158 | 0.117 | 0.213 | 0.573 | 0.113 | 0.291 |
| 0.562 | 0.278 | 0.930 | 0.453 | 1.000 | 0.113 | 0.152 | 0.110 | 0.215 | 0.595 | 0.130 | 0.269 |
| 0.556 | 0.246 | 0.920 | 0.490 | 0.900 | 0.104 | 0.147 | 0.417 | 0.209 | 0.528 | 0.119 | 0.245 |
| 0.658 | 0.273 | 0.980 | 0.434 | 0.910 | 0.124 | 0.155 | 0.112 | 0.193 | 0.567 | 0.124 | 0.286 |
| 0.610 | 0.248 | 0.940 | 0.420 | 1.000 | 0.115 | 0.139 | 0.102 | 0.189 | 0.506 | 0.124 | 0.280 |
| 0.550 | 0.265 | 0.980 | 0.448 | 1.000 | 0.106 | 0.139 | 0.111 | 0.201 | 0.573 | 0.133 | 0.267 |
| 0.562 | 0.286 | 1.000 | 0.480 | 1.000 | 0.103 | 0.138 | 0.114 | 0.203 | 0.550 | 0.134 | 0.253 |
| 0.628 | 0.265 | 1.000 | 0.453 | 1.000 | 0.103 | 0.145 | 0.116 | 0.203 | 0.600 | 0.117 | 0.250 |
| 0.592 | 0.265 | 1.000 | 0.467 | 1.000 | 0.115 | 0.151 | 0.096 | 0.199 | 0.578 | 0.128 | 0.275 |
| 0.664 | 0.281 | 1.000 | 0.457 | 1.000 | 0.124 | 0.136 | 0.101 | 0.183 | 0.573 | 0.133 | 0.256 |
| 0.598 | 0.297 | 1.000 | 0.508 | 1.000 | 0.119 | 0.157 | 0.117 | 0.197 | 0.500 | 0.111 | 0.286 |
| 0.616 | 0.243 | 1.000 | 0.453 | 1.000 | 0.102 | 0.157 | 0.103 | 0.193 | 0.517 | 0.119 | 0.277 |
| 0.622 | 0.270 | 1.000 | 0.425 | 1.000 | 0.120 | 0.142 | 0.102 | 0.201 | 0.573 | 0.112 | 0.283 |
| 0.640 | 0.292 | 1.000 | 0.453 | 1.000 | 0.121 | 0.149 | 0.112 | 0.183 | 0.539 | 0.135 | 0.253 |
| 0.568 | 0.270 | 0.990 | 0.499 | 0.910 | 0.121 | 0.163 | 0.104 | 0.217 | 0.595 | 0.119 | 0.248 |
| 0.580 | 0.286 | 0.920 | 0.448 | 1.000 | 0.102 | 0.141 | 0.103 | 0.213 | 0.573 | 0.132 | 0.250 |
| 0.634 | 0.289 | 0.940 | 0.444 | 1.000 | 0.105 | 0.151 | 0.117 | 0.191 | 0.589 | 0.117 | 0.277 |
| 0.556 | 0.294 | 0.950 | 0.467 | 1.000 | 0.107 | 0.154 | 0.108 | 0.211 | 0.606 | 0.128 | 0.258 |
| 0.568 | 0.286 | 0.960 | 0.453 | 1.000 | 0.113 | 0.144 | 0.117 | 0.219 | 0.612 | 0.117 | 0.283 |
| 0.646 | 0.243 | 0.980 | 0.453 | 1.000 | 0.113 | 0.151 | 0.100 | 0.195 | 0.512 | 0.132 | 0.294 |
| 0.592 | 0.254 | 0.980 | 0.416 | 1.000 | 0.107 | 0.142 | 0.104 | 0.195 | 0.545 | 0.112 | 0.261 |
| 0.622 | 0.248 | 0.990 | 0.420 | 1.000 | 0.114 | 0.136 | 0.113 | 0.203 | 0.534 | 0.133 | 0.250 |
| 0.646 | 0.248 | 0.990 | 0.462 | 1.000 | 0.104 | 0.151 | 0.112 | 0.197 | 0.584 | 0.117 | 0.245 |
| 0.580 | 0.281 | 0.990 | 0.499 | 1.000 | 0.113 | 0.133 | 0.110 | 0.193 | 0.573 | 0.133 | 0.280 |
| 0.544 | 0.284 | 1.000 | 0.490 | 1.000 | 0.120 | 0.148 | 0.110 | 0.197 | 0.523 | 0.132 | 0.253 |
| 0.562 | 0.248 | 0.990 | 0.508 | 0.910 | 0.114 | 0.163 | 0.112 | 0.205 | 0.523 | 0.125 | 0.269 |
| 0.544 | 0.270 | 1.000 | 0.430 | 1.000 | 0.103 | 0.155 | 0.098 | 0.197 | 0.517 | 0.117 | 0.253 |
| 0.610 | 0.251 | 1.000 | 0.434 | 1.000 | 0.104 | 0.147 | 0.096 | 0.191 | 0.567 | 0.130 | 0.261 |
| 0.652 | 0.265 | 1.000 | 0.457 | 1.000 | 0.116 | 0.139 | 0.109 | 0.213 | 0.523 | 0.117 | 0.294 |
| 0.586 | 0.278 | 1.000 | 0.434 | 1.000 | 0.104 | 0.157 | 0.104 | 0.187 | 0.545 | 0.128 | 0.245 |
| 0.598 | 0.265 | 1.000 | 0.499 | 1.000 | 0.107 | 0.155 | 0.105 | 0.185 | 0.528 | 0.134 | 0.245 |
| 0.544 | 0.257 | 1.000 | 0.425 | 1.000 | 0.116 | 0.147 | 0.109 | 0.185 | 0.556 | 0.124 | 0.280 |
| 0.574 | 0.262 | 1.000 | 0.416 | 1.000 | 0.121 | 0.158 | 0.114 | 0.181 | 0.562 | 0.118 | 0.299 |
| 0.550 | 0.270 | 1.000 | 0.504 | 1.000 | 0.121 | 0.138 | 0.100 | 0.201 | 0.562 | 0.125 | 0.272 |
| 0.562 | 0.243 | 1.000 | 0.420 | 1.000 | 0.105 | 0.135 | 0.101 | 0.213 | 0.506 | 0.130 | 0.256 |
| 0.562 | 0.251 | 1.000 | 0.490 | 1.000 | 0.102 | 0.152 | 0.101 | 0.185 | 0.523 | 0.135 | 0.261 |
| 0.640 | 0.275 | 0.990 | 0.420 | 0.910 | 0.123 | 0.147 | 0.112 | 0.197 | 0.517 | 0.311 | 0.272 |
| 0.640 | 0.292 | 0.900 | 0.490 | 1.000 | 0.118 | 0.158 | 0.106 | 0.211 | 0.589 | 0.121 | 0.264 |
| 0.616 | 0.289 | 0.900 | 0.504 | 1.000 | 0.113 | 0.139 | 0.102 | 0.191 | 0.573 | 0.111 | 0.277 |
| 0.652 | 0.246 | 0.910 | 0.467 | 1.000 | 0.118 | 0.144 | 0.100 | 0.189 | 0.500 | 0.111 | 0.267 |
| 0.658 | 0.286 | 0.930 | 0.430 | 1.000 | 0.119 | 0.138 | 0.101 | 0.213 | 0.512 | 0.134 | 0.245 |
| 0.616 | 0.292 | 0.940 | 0.434 | 1.000 | 0.108 | 0.135 | 0.118 | 0.195 | 0.589 | 0.114 | 0.250 |
| 0.586 | 0.294 | 0.940 | 0.439 | 1.000 | 0.114 | 0.142 | 0.104 | 0.209 | 0.612 | 0.116 | 0.286 |
| 0.616 | 0.243 | 0.960 | 0.462 | 1.000 | 0.123 | 0.163 | 0.100 | 0.215 | 0.528 | 0.129 | 0.250 |
| 0.586 | 0.286 | 0.960 | 0.430 | 1.000 | 0.121 | 0.155 | 0.100 | 0.211 | 0.573 | 0.111 | 0.286 |
| 0.658 | 0.262 | 0.960 | 0.467 | 1.000 | 0.110 | 0.155 | 0.111 | 0.215 | 0.517 | 0.130 | 0.253 |
| 0.616 | 0.270 | 0.980 | 0.453 | 1.000 | 0.107 | 0.136 | 0.111 | 0.217 | 0.589 | 0.118 | 0.294 |
| 0.628 | 0.251 | 1.000 | 0.462 | 0.910 | 0.103 | 0.154 | 0.114 | 0.185 | 0.612 | 0.114 | 0.277 |
| 0.586 | 0.294 | 0.980 | 0.467 | 1.000 | 0.123 | 0.139 | 0.100 | 0.211 | 0.550 | 0.121 | 0.291 |
| 0.616 | 0.243 | 1.000 | 0.504 | 1.000 | 0.110 | 0.160 | 0.105 | 0.205 | 0.573 | 0.132 | 0.267 |
| 0.574 | 0.294 | 1.000 | 0.453 | 1.000 | 0.112 | 0.145 | 0.104 | 0.183 | 0.545 | 0.127 | 0.299 |
| 0.592 | 0.284 | 1.000 | 0.439 | 1.000 | 0.114 | 0.138 | 0.100 | 0.201 | 0.589 | 0.112 | 0.250 |
| 0.658 | 0.284 | 1.000 | 0.420 | 1.000 | 0.110 | 0.152 | 0.111 | 0.185 | 0.562 | 0.132 | 0.256 |
| 0.652 | 0.275 | 1.000 | 0.453 | 1.000 | 0.116 | 0.161 | 0.113 | 0.189 | 0.550 | 0.125 | 0.299 |
| 0.646 | 0.270 | 1.000 | 0.420 | 1.000 | 0.111 | 0.145 | 0.113 | 0.187 | 0.550 | 0.116 | 0.275 |
| 0.556 | 0.262 | 1.000 | 0.462 | 1.000 | 0.102 | 0.161 | 0.108 | 0.185 | 0.556 | 0.114 | 0.258 |
| 0.574 | 0.284 | 1.000 | 0.416 | 1.000 | 0.104 | 0.163 | 0.106 | 0.201 | 0.606 | 0.122 | 0.245 |
| 0.658 | 0.257 | 1.000 | 0.508 | 1.000 | 0.123 | 0.152 | 0.113 | 0.191 | 0.523 | 0.123 | 0.253 |
| 0.610 | 0.246 | 1.000 | 0.490 | 0.910 | 0.116 | 0.135 | 0.411 | 0.179 | 0.534 | 0.112 | 0.277 |
| 0.586 | 0.246 | 1.000 | 0.425 | 1.000 | 0.119 | 0.145 | 0.117 | 0.189 | 0.562 | 0.134 | 0.258 |
| 0.616 | 0.246 | 1.000 | 0.490 | 1.000 | 0.102 | 0.158 | 0.102 | 0.179 | 0.595 | 0.127 | 0.258 |
| 0.664 | 0.286 | 0.900 | 0.430 | 1.000 | 0.107 | 0.148 | 0.110 | 0.189 | 0.528 | 0.114 | 0.256 |
| 0.562 | 0.243 | 0.920 | 0.439 | 1.000 | 0.107 | 0.161 | 0.112 | 0.219 | 0.550 | 0.135 | 0.269 |
| 0.664 | 0.286 | 0.920 | 0.448 | 1.000 | 0.123 | 0.161 | 0.105 | 0.195 | 0.600 | 0.135 | 0.256 |
| 0.664 | 0.254 | 0.930 | 0.444 | 1.000 | 0.113 | 0.157 | 0.107 | 0.187 | 0.545 | 0.135 | 0.269 |
| 0.634 | 0.251 | 0.940 | 0.439 | 1.000 | 0.107 | 0.135 | 0.113 | 0.185 | 0.573 | 0.130 | 0.286 |
| 0.568 | 0.273 | 0.960 | 0.425 | 1.000 | 0.114 | 0.158 | 0.097 | 0.193 | 0.556 | 0.114 | 0.288 |
| 0.610 | 0.254 | 0.960 | 0.480 | 1.000 | 0.105 | 0.158 | 0.097 | 0.191 | 0.539 | 0.124 | 0.288 |
| 0.652 | 0.257 | 0.960 | 0.439 | 1.000 | 0.112 | 0.136 | 0.102 | 0.189 | 0.506 | 0.132 | 0.258 |
| 0.628 | 0.289 | 1.000 | 0.476 | 0.910 | 0.113 | 0.152 | 0.310 | 0.195 | 0.556 | 0.129 | 0.294 |
| 0.580 | 0.262 | 0.970 | 0.439 | 1.000 | 0.123 | 0.142 | 0.118 | 0.211 | 0.506 | 0.124 | 0.299 |
| 0.652 | 0.294 | 0.970 | 0.490 | 1.000 | 0.106 | 0.145 | 0.103 | 0.215 | 0.589 | 0.134 | 0.288 |
| 0.658 | 0.251 | 0.970 | 0.504 | 1.000 | 0.110 | 0.158 | 0.097 | 0.199 | 0.589 | 0.113 | 0.294 |
| 0.634 | 0.294 | 0.980 | 0.476 | 1.000 | 0.121 | 0.160 | 0.111 | 0.189 | 0.539 | 0.132 | 0.264 |
| 0.556 | 0.289 | 0.990 | 0.504 | 1.000 | 0.123 | 0.163 | 0.111 | 0.183 | 0.612 | 0.113 | 0.250 |
| 0.550 | 0.270 | 1.000 | 0.485 | 1.000 | 0.115 | 0.155 | 0.118 | 0.193 | 0.589 | 0.122 | 0.267 |
| 0.622 | 0.297 | 1.000 | 0.457 | 1.000 | 0.104 | 0.161 | 0.106 | 0.211 | 0.550 | 0.114 | 0.296 |
| 0.592 | 0.284 | 1.000 | 0.467 | 1.000 | 0.121 | 0.148 | 0.104 | 0.219 | 0.500 | 0.123 | 0.261 |
| 0.628 | 0.273 | 1.000 | 0.504 | 1.000 | 0.114 | 0.133 | 0.101 | 0.211 | 0.528 | 0.116 | 0.248 |
| 0.616 | 0.281 | 1.000 | 0.416 | 1.000 | 0.113 | 0.135 | 0.102 | 0.191 | 0.517 | 0.135 | 0.250 |
| 0.768 | 0.246 | 1.000 | 0.434 | 0.910 | 0.123 | 0.161 | 0.210 | 0.213 | 0.512 | 0.132 | 0.296 |
| 0.628 | 0.267 | 1.000 | 0.508 | 1.000 | 0.114 | 0.152 | 0.109 | 0.195 | 0.562 | 0.134 | 0.258 |
| 0.556 | 0.273 | 1.000 | 0.430 | 1.000 | 0.104 | 0.152 | 0.096 | 0.179 | 0.506 | 0.116 | 0.280 |
| 0.586 | 0.257 | 0.900 | 0.430 | 1.000 | 0.122 | 0.161 | 0.109 | 0.213 | 0.523 | 0.125 | 0.272 |
| 0.556 | 0.243 | 0.900 | 0.476 | 1.000 | 0.114 | 0.148 | 0.112 | 0.205 | 0.517 | 0.135 | 0.275 |
| 0.550 | 0.275 | 0.910 | 0.439 | 1.000 | 0.102 | 0.139 | 0.107 | 0.195 | 0.500 | 0.122 | 0.275 |
| 0.580 | 0.254 | 0.910 | 0.485 | 1.000 | 0.118 | 0.155 | 0.117 | 0.211 | 0.612 | 0.135 | 0.277 |
| 0.592 | 0.292 | 0.910 | 0.462 | 1.000 | 0.118 | 0.155 | 0.110 | 0.185 | 0.506 | 0.114 | 0.250 |
| 0.604 | 0.243 | 0.930 | 0.434 | 1.000 | 0.106 | 0.149 | 0.098 | 0.183 | 0.562 | 0.116 | 0.245 |
| 0.562 | 0.248 | 0.940 | 0.430 | 1.000 | 0.113 | 0.149 | 0.101 | 0.195 | 0.550 | 0.127 | 0.267 |
| 0.550 | 0.251 | 0.950 | 0.508 | 1.000 | 0.106 | 0.149 | 0.116 | 0.191 | 0.506 | 0.124 | 0.253 |
| 0.658 | 0.259 | 1.000 | 0.434 | 0.910 | 0.104 | 0.157 | 0.510 | 0.207 | 0.523 | 0.312 | 0.248 |
| 0.646 | 0.281 | 0.960 | 0.476 | 1.000 | 0.106 | 0.133 | 0.096 | 0.181 | 0.562 | 0.119 | 0.248 |
| 0.628 | 0.267 | 0.970 | 0.480 | 1.000 | 0.123 | 0.154 | 0.096 | 0.213 | 0.523 | 0.128 | 0.253 |
| 0.562 | 0.275 | 0.970 | 0.485 | 1.000 | 0.118 | 0.139 | 0.102 | 0.187 | 0.528 | 0.119 | 0.286 |
| 0.622 | 0.251 | 0.980 | 0.425 | 1.000 | 0.113 | 0.133 | 0.108 | 0.187 | 0.506 | 0.127 | 0.288 |
| 0.640 | 0.289 | 0.990 | 0.439 | 1.000 | 0.108 | 0.148 | 0.113 | 0.187 | 0.600 | 0.119 | 0.245 |
| 0.580 | 0.297 | 1.000 | 0.434 | 1.000 | 0.102 | 0.142 | 0.117 | 0.189 | 0.595 | 0.113 | 0.269 |
| 0.628 | 0.262 | 1.000 | 0.504 | 1.000 | 0.124 | 0.147 | 0.107 | 0.209 | 0.506 | 0.114 | 0.272 |
| 0.616 | 0.265 | 1.000 | 0.448 | 1.000 | 0.111 | 0.151 | 0.109 | 0.191 | 0.506 | 0.121 | 0.258 |
| 0.550 | 0.278 | 1.000 | 0.434 | 1.000 | 0.107 | 0.155 | 0.117 | 0.179 | 0.578 | 0.121 | 0.258 |
| 0.622 | 0.294 | 1.000 | 0.430 | 1.000 | 0.112 | 0.158 | 0.097 | 0.199 | 0.562 | 0.114 | 0.248 |
| 0.568 | 0.275 | 1.000 | 0.480 | 0.910 | 0.121 | 0.149 | 0.118 | 0.193 | 0.550 | 0.122 | 0.294 |
| 0.610 | 0.294 | 1.000 | 0.467 | 1.000 | 0.124 | 0.158 | 0.103 | 0.197 | 0.589 | 0.133 | 0.267 |
| 0.604 | 0.292 | 1.000 | 0.457 | 1.000 | 0.111 | 0.152 | 0.102 | 0.195 | 0.550 | 0.116 | 0.291 |
| 0.640 | 0.259 | 1.000 | 0.467 | 1.000 | 0.124 | 0.136 | 0.102 | 0.187 | 0.612 | 0.122 | 0.272 |
| 0.592 | 0.243 | 1.000 | 0.434 | 1.000 | 0.124 | 0.133 | 0.098 | 0.205 | 0.523 | 0.133 | 0.275 |
| 0.574 | 0.270 | 1.000 | 0.467 | 1.000 | 0.102 | 0.149 | 0.102 | 0.219 | 0.612 | 0.113 | 0.280 |
| 0.592 | 0.270 | 0.900 | 0.434 | 1.000 | 0.105 | 0.145 | 0.108 | 0.203 | 0.523 | 0.125 | 0.245 |
| 0.586 | 0.265 | 0.910 | 0.430 | 1.000 | 0.114 | 0.135 | 0.098 | 0.207 | 0.528 | 0.123 | 0.267 |
| 0.658 | 0.289 | 0.910 | 0.430 | 1.000 | 0.104 | 0.154 | 0.103 | 0.187 | 0.556 | 0.118 | 0.253 |
| 0.616 | 0.284 | 0.950 | 0.453 | 1.000 | 0.111 | 0.139 | 0.103 | 0.189 | 0.528 | 0.122 | 0.269 |
| 0.640 | 0.289 | 0.950 | 0.508 | 1.000 | 0.110 | 0.133 | 0.102 | 0.215 | 0.539 | 0.123 | 0.269 |
| 0.616 | 0.275 | 0.920 | 0.416 | 0.900 | 0.123 | 0.133 | 0.102 | 0.183 | 0.595 | 0.111 | 0.256 |
| 0.568 | 0.267 | 1.000 | 0.494 | 0.910 | 0.104 | 0.158 | 0.098 | 0.219 | 0.606 | 0.123 | 0.283 |
| 0.604 | 0.297 | 0.950 | 0.457 | 1.000 | 0.110 | 0.155 | 0.097 | 0.219 | 0.606 | 0.119 | 0.253 |
| 0.556 | 0.265 | 0.950 | 0.508 | 1.000 | 0.115 | 0.141 | 0.096 | 0.189 | 0.506 | 0.130 | 0.272 |
| 0.550 | 0.292 | 0.960 | 0.508 | 1.000 | 0.115 | 0.136 | 0.117 | 0.207 | 0.528 | 0.129 | 0.253 |
| 0.574 | 0.243 | 0.960 | 0.471 | 1.000 | 0.118 | 0.157 | 0.097 | 0.201 | 0.550 | 0.119 | 0.267 |
| 0.634 | 0.281 | 0.960 | 0.471 | 1.000 | 0.115 | 0.138 | 0.103 | 0.185 | 0.567 | 0.114 | 0.256 |
| 0.580 | 0.273 | 0.990 | 0.462 | 1.000 | 0.310 | 0.151 | 0.114 | 0.181 | 0.595 | 0.125 | 0.245 |
| 0.592 | 0.270 | 0.990 | 0.499 | 1.000 | 0.113 | 0.151 | 0.101 | 0.187 | 0.595 | 0.113 | 0.248 |
| 0.616 | 0.254 | 1.000 | 0.439 | 1.000 | 0.220 | 0.136 | 0.107 | 0.187 | 0.584 | 0.128 | 0.264 |
| 0.610 | 0.243 | 1.000 | 0.485 | 1.000 | 0.106 | 0.148 | 0.102 | 0.219 | 0.539 | 0.128 | 0.272 |
| 0.556 | 0.243 | 1.000 | 0.453 | 1.000 | 0.102 | 0.161 | 0.103 | 0.195 | 0.578 | 0.133 | 0.258 |
| 0.622 | 0.289 | 1.000 | 0.448 | 0.910 | 0.115 | 0.135 | 0.117 | 0.187 | 0.573 | 0.113 | 0.288 |
| 0.652 | 0.267 | 1.000 | 0.504 | 1.000 | 0.116 | 0.144 | 0.105 | 0.209 | 0.578 | 0.125 | 0.294 |
| 0.568 | 0.254 | 1.000 | 0.457 | 1.000 | 0.107 | 0.154 | 0.104 | 0.205 | 0.556 | 0.123 | 0.286 |
| 0.658 | 0.292 | 1.000 | 0.480 | 1.000 | 0.103 | 0.160 | 0.102 | 0.201 | 0.512 | 0.119 | 0.256 |
| 0.640 | 0.270 | 1.000 | 0.430 | 1.000 | 0.115 | 0.155 | 0.112 | 0.189 | 0.545 | 0.123 | 0.261 |
| 0.604 | 0.292 | 1.000 | 0.490 | 1.000 | 0.119 | 0.149 | 0.112 | 0.197 | 0.573 | 0.114 | 0.294 |
| 0.652 | 0.281 | 1.000 | 0.457 | 1.000 | 0.119 | 0.139 | 0.117 | 0.207 | 0.512 | 0.128 | 0.299 |
| 0.556 | 0.248 | 1.000 | 0.420 | 1.000 | 0.123 | 0.160 | 0.114 | 0.213 | 0.517 | 0.130 | 0.299 |
| 0.664 | 0.275 | 1.000 | 0.485 | 1.000 | 0.119 | 0.139 | 0.110 | 0.215 | 0.589 | 0.114 | 0.277 |
| 0.568 | 0.289 | 1.000 | 0.467 | 1.000 | 0.105 | 0.145 | 0.111 | 0.213 | 0.528 | 0.134 | 0.256 |
| 0.610 | 0.273 | 0.900 | 0.508 | 1.000 | 0.122 | 0.138 | 0.098 | 0.201 | 0.506 | 0.125 | 0.256 |
| 0.556 | 0.273 | 1.000 | 0.425 | 0.910 | 0.106 | 0.155 | 0.110 | 0.187 | 0.612 | 0.117 | 0.299 |
| 0.562 | 0.292 | 0.910 | 0.416 | 1.000 | 0.119 | 0.147 | 0.114 | 0.179 | 0.589 | 0.129 | 0.277 |
| 0.556 | 0.270 | 0.910 | 0.462 | 1.000 | 0.103 | 0.144 | 0.118 | 0.213 | 0.562 | 0.113 | 0.275 |
| 0.664 | 0.286 | 0.920 | 0.494 | 1.000 | 0.124 | 0.154 | 0.114 | 0.211 | 0.562 | 0.117 | 0.272 |
| 0.598 | 0.243 | 0.920 | 0.499 | 1.000 | 0.124 | 0.163 | 0.096 | 0.197 | 0.545 | 0.134 | 0.277 |
| 0.592 | 0.286 | 0.930 | 0.494 | 1.000 | 0.105 | 0.138 | 0.102 | 0.217 | 0.612 | 0.133 | 0.288 |
| 0.556 | 0.284 | 0.940 | 0.430 | 1.000 | 0.106 | 0.154 | 0.105 | 0.197 | 0.539 | 0.111 | 0.245 |
| 0.556 | 0.284 | 0.940 | 0.430 | 1.000 | 0.111 | 0.151 | 0.103 | 0.213 | 0.506 | 0.121 | 0.272 |
| 0.592 | 0.251 | 0.950 | 0.471 | 1.000 | 0.114 | 0.155 | 0.111 | 0.199 | 0.545 | 0.134 | 0.286 |
| 0.562 | 0.284 | 0.950 | 0.467 | 1.000 | 0.106 | 0.148 | 0.110 | 0.207 | 0.556 | 0.117 | 0.283 |
| 0.652 | 0.265 | 0.970 | 0.430 | 1.000 | 0.116 | 0.149 | 0.110 | 0.205 | 0.606 | 0.129 | 0.267 |
| 0.610 | 0.270 | 1.000 | 0.471 | 0.910 | 0.124 | 0.145 | 0.109 | 0.181 | 0.517 | 0.117 | 0.272 |
| 0.586 | 0.248 | 0.970 | 0.499 | 1.000 | 0.111 | 0.148 | 0.118 | 0.185 | 0.523 | 0.122 | 0.253 |
| 0.610 | 0.273 | 0.970 | 0.444 | 1.000 | 0.107 | 0.149 | 0.103 | 0.195 | 0.573 | 0.114 | 0.253 |
| 0.574 | 0.273 | 0.970 | 0.485 | 1.000 | 0.103 | 0.155 | 0.096 | 0.209 | 0.517 | 0.118 | 0.269 |
| 0.604 | 0.270 | 0.980 | 0.471 | 1.000 | 0.102 | 0.158 | 0.108 | 0.199 | 0.556 | 0.112 | 0.261 |
| 0.664 | 0.292 | 0.980 | 0.425 | 1.000 | 0.123 | 0.161 | 0.112 | 0.195 | 0.589 | 0.122 | 0.253 |
| 0.622 | 0.262 | 1.000 | 0.490 | 1.000 | 0.102 | 0.138 | 0.104 | 0.181 | 0.584 | 0.129 | 0.283 |
| 0.646 | 0.251 | 1.000 | 0.499 | 1.000 | 0.113 | 0.142 | 0.105 | 0.185 | 0.534 | 0.132 | 0.288 |
| 0.556 | 0.265 | 1.000 | 0.416 | 1.000 | 0.106 | 0.133 | 0.108 | 0.183 | 0.578 | 0.130 | 0.291 |
| 0.580 | 0.243 | 1.000 | 0.457 | 1.000 | 0.103 | 0.157 | 0.111 | 0.187 | 0.512 | 0.125 | 0.291 |
| 0.664 | 0.275 | 1.000 | 0.425 | 1.000 | 0.107 | 0.144 | 0.106 | 0.199 | 0.550 | 0.121 | 0.277 |
| 0.652 | 0.270 | 1.000 | 0.453 | 0.910 | 0.122 | 0.139 | 0.112 | 0.179 | 0.578 | 0.134 | 0.258 |
| 0.652 | 0.262 | 1.000 | 0.494 | 1.000 | 0.124 | 0.144 | 0.118 | 0.195 | 0.534 | 0.122 | 0.256 |
| 0.556 | 0.297 | 1.000 | 0.420 | 1.000 | 0.107 | 0.148 | 0.107 | 0.209 | 0.539 | 0.128 | 0.264 |
| 0.586 | 0.262 | 1.000 | 0.420 | 1.000 | 0.110 | 0.161 | 0.110 | 0.207 | 0.506 | 0.124 | 0.283 |
| 0.592 | 0.286 | 1.000 | 0.448 | 1.000 | 0.123 | 0.147 | 0.112 | 0.195 | 0.506 | 0.112 | 0.299 |
| 0.616 | 0.259 | 0.930 | 0.444 | 1.000 | 0.111 | 0.145 | 0.105 | 0.191 | 0.606 | 0.124 | 0.291 |
| 0.556 | 0.292 | 0.940 | 0.444 | 1.000 | 0.114 | 0.157 | 0.112 | 0.195 | 0.573 | 0.116 | 0.248 |
| 0.586 | 0.262 | 0.940 | 0.467 | 1.000 | 0.111 | 0.147 | 0.114 | 0.217 | 0.534 | 0.122 | 0.245 |
| 0.550 | 0.262 | 0.950 | 0.480 | 1.000 | 0.106 | 0.155 | 0.106 | 0.217 | 0.517 | 0.123 | 0.299 |
| 0.628 | 0.286 | 0.960 | 0.504 | 1.000 | 0.119 | 0.152 | 0.108 | 0.193 | 0.612 | 0.121 | 0.275 |
| 0.610 | 0.243 | 0.970 | 0.494 | 1.000 | 0.115 | 0.163 | 0.098 | 0.193 | 0.589 | 0.114 | 0.294 |
| 0.592 | 0.284 | 1.000 | 0.480 | 0.910 | 0.110 | 0.163 | 0.109 | 0.207 | 0.584 | 0.135 | 0.250 |
| 0.556 | 0.257 | 0.970 | 0.485 | 1.000 | 0.107 | 0.160 | 0.113 | 0.209 | 0.534 | 0.125 | 0.291 |
| 0.610 | 0.259 | 0.970 | 0.444 | 1.000 | 0.111 | 0.163 | 0.103 | 0.191 | 0.523 | 0.127 | 0.245 |
| 0.562 | 0.286 | 0.980 | 0.462 | 1.000 | 0.105 | 0.158 | 0.118 | 0.203 | 0.523 | 0.127 | 0.245 |
| 0.586 | 0.251 | 0.990 | 0.462 | 1.000 | 0.108 | 0.145 | 0.111 | 0.181 | 0.556 | 0.132 | 0.275 |
| 0.646 | 0.286 | 1.000 | 0.490 | 1.000 | 0.102 | 0.148 | 0.096 | 0.209 | 0.595 | 0.122 | 0.258 |
| 0.664 | 0.297 | 1.000 | 0.420 | 1.000 | 0.114 | 0.161 | 0.101 | 0.213 | 0.589 | 0.134 | 0.258 |
| 0.580 | 0.297 | 1.000 | 0.499 | 1.000 | 0.114 | 0.161 | 0.112 | 0.185 | 0.523 | 0.117 | 0.296 |
| 0.610 | 0.284 | 1.000 | 0.444 | 1.000 | 0.122 | 0.135 | 0.114 | 0.179 | 0.545 | 0.123 | 0.264 |
| 0.640 | 0.270 | 1.000 | 0.425 | 1.000 | 0.116 | 0.141 | 0.111 | 0.207 | 0.567 | 0.113 | 0.286 |
| 0.568 | 0.275 | 1.000 | 0.425 | 1.000 | 0.121 | 0.136 | 0.102 | 0.209 | 0.550 | 0.128 | 0.267 |
| 0.634 | 0.278 | 1.000 | 0.448 | 0.910 | 0.102 | 0.148 | 0.101 | 0.201 | 0.595 | 0.121 | 0.248 |
| 0.544 | 0.294 | 1.000 | 0.499 | 1.000 | 0.122 | 0.161 | 0.098 | 0.189 | 0.545 | 0.111 | 0.272 |
| 0.592 | 0.257 | 1.000 | 0.430 | 1.000 | 0.113 | 0.139 | 0.107 | 0.203 | 0.556 | 0.125 | 0.250 |
| 0.586 | 0.284 | 1.000 | 0.439 | 1.000 | 0.113 | 0.152 | 0.112 | 0.189 | 0.556 | 0.117 | 0.280 |
| 0.762 | 0.257 | 1.000 | 0.490 | 1.000 | 0.118 | 0.161 | 0.106 | 0.199 | 0.573 | 0.122 | 0.277 |
| 0.658 | 0.289 | 1.000 | 0.490 | 1.000 | 0.104 | 0.141 | 0.102 | 0.219 | 0.528 | 0.124 | 0.283 |
| 0.574 | 0.294 | 1.000 | 0.457 | 1.000 | 0.106 | 0.145 | 0.113 | 0.199 | 0.500 | 0.133 | 0.258 |
| 0.610 | 0.259 | 1.000 | 0.494 | 1.000 | 0.105 | 0.155 | 0.109 | 0.179 | 0.556 | 0.128 | 0.261 |
| 0.658 | 0.292 | 1.000 | 0.434 | 1.000 | 0.105 | 0.142 | 0.100 | 0.201 | 0.545 | 0.117 | 0.267 |
| 0.598 | 0.273 | 1.000 | 0.508 | 1.000 | 0.115 | 0.154 | 0.098 | 0.213 | 0.562 | 0.112 | 0.286 |
| 0.646 | 0.284 | 1.000 | 0.448 | 1.000 | 0.124 | 0.136 | 0.102 | 0.199 | 0.573 | 0.123 | 0.280 |
| 0.604 | 0.278 | 1.000 | 0.508 | 0.910 | 0.108 | 0.157 | 0.112 | 0.197 | 0.612 | 0.118 | 0.256 |
| 0.580 | 0.289 | 0.910 | 0.494 | 1.000 | 0.122 | 0.155 | 0.111 | 0.199 | 0.578 | 0.132 | 0.280 |
| 0.740 | 0.273 | 0.910 | 0.439 | 1.000 | 0.105 | 0.144 | 0.111 | 0.179 | 0.500 | 0.129 | 0.280 |
| 0.646 | 0.289 | 0.920 | 0.444 | 1.000 | 0.103 | 0.145 | 0.111 | 0.219 | 0.545 | 0.132 | 0.275 |
| 0.604 | 0.281 | 0.920 | 0.457 | 1.000 | 0.122 | 0.155 | 0.105 | 0.203 | 0.545 | 0.116 | 0.258 |
| 0.640 | 0.297 | 0.930 | 0.416 | 1.000 | 0.110 | 0.138 | 0.111 | 0.191 | 0.512 | 0.132 | 0.299 |
| 0.658 | 0.259 | 0.940 | 0.457 | 1.000 | 0.113 | 0.152 | 0.110 | 0.183 | 0.567 | 0.128 | 0.288 |
| 0.628 | 0.248 | 0.960 | 0.467 | 1.000 | 0.103 | 0.145 | 0.111 | 0.201 | 0.600 | 0.135 | 0.258 |
| 0.556 | 0.297 | 0.970 | 0.494 | 1.000 | 0.106 | 0.161 | 0.104 | 0.197 | 0.606 | 0.135 | 0.272 |
| 0.646 | 0.273 | 0.970 | 0.480 | 1.000 | 0.112 | 0.148 | 0.111 | 0.207 | 0.595 | 0.135 | 0.291 |
| 0.580 | 0.267 | 0.970 | 0.485 | 1.000 | 0.116 | 0.138 | 0.118 | 0.205 | 0.567 | 0.111 | 0.280 |
| 0.550 | 0.297 | 1.000 | 0.494 | 0.910 | 0.122 | 0.154 | 0.096 | 0.183 | 0.578 | 0.311 | 0.280 |
| 0.550 | 0.257 | 0.980 | 0.453 | 1.000 | 0.124 | 0.158 | 0.105 | 0.215 | 0.562 | 0.112 | 0.299 |
| 0.592 | 0.248 | 0.980 | 0.471 | 1.000 | 0.122 | 0.141 | 0.117 | 0.219 | 0.512 | 0.116 | 0.267 |
| 0.780 | 0.257 | 0.990 | 0.453 | 1.000 | 0.116 | 0.141 | 0.103 | 0.199 | 0.606 | 0.124 | 0.272 |
| 0.658 | 0.294 | 1.000 | 0.425 | 1.000 | 0.121 | 0.136 | 0.106 | 0.187 | 0.534 | 0.113 | 0.272 |
| 0.616 | 0.254 | 1.000 | 0.471 | 1.000 | 0.106 | 0.135 | 0.096 | 0.217 | 0.578 | 0.118 | 0.269 |
| 0.628 | 0.289 | 1.000 | 0.457 | 1.000 | 0.115 | 0.158 | 0.107 | 0.207 | 0.539 | 0.123 | 0.250 |
| 0.556 | 0.270 | 1.000 | 0.480 | 1.000 | 0.118 | 0.148 | 0.114 | 0.197 | 0.517 | 0.122 | 0.286 |
| 0.586 | 0.251 | 1.000 | 0.476 | 1.000 | 0.124 | 0.161 | 0.106 | 0.205 | 0.584 | 0.134 | 0.248 |
| 0.658 | 0.294 | 1.000 | 0.471 | 1.000 | 0.116 | 0.147 | 0.103 | 0.215 | 0.589 | 0.117 | 0.250 |
| 0.646 | 0.267 | 1.000 | 0.480 | 1.000 | 0.120 | 0.144 | 0.107 | 0.199 | 0.528 | 0.118 | 0.280 |
| 0.604 | 0.262 | 1.000 | 0.457 | 0.910 | 0.108 | 0.142 | 0.098 | 0.195 | 0.556 | 0.125 | 0.294 |
| 0.610 | 0.294 | 1.000 | 0.508 | 1.000 | 0.120 | 0.144 | 0.114 | 0.187 | 0.589 | 0.134 | 0.245 |
| 0.640 | 0.265 | 1.000 | 0.467 | 1.000 | 0.120 | 0.135 | 0.116 | 0.219 | 0.534 | 0.130 | 0.250 |
| 0.664 | 0.267 | 1.000 | 0.462 | 1.000 | 0.102 | 0.148 | 0.118 | 0.199 | 0.589 | 0.127 | 0.275 |
| 0.658 | 0.297 | 1.000 | 0.471 | 1.000 | 0.107 | 0.145 | 0.096 | 0.199 | 0.512 | 0.113 | 0.277 |
| 0.616 | 0.246 | 1.000 | 0.499 | 1.000 | 0.123 | 0.154 | 0.098 | 0.179 | 0.528 | 0.127 | 0.253 |
| 0.580 | 0.294 | 1.000 | 0.490 | 1.000 | 0.106 | 0.135 | 0.111 | 0.189 | 0.556 | 0.112 | 0.248 |
| 0.634 | 0.262 | 1.000 | 0.467 | 1.000 | 0.115 | 0.160 | 0.114 | 0.191 | 0.506 | 0.119 | 0.280 |
| 0.568 | 0.292 | 1.000 | 0.453 | 1.000 | 0.122 | 0.157 | 0.104 | 0.185 | 0.584 | 0.123 | 0.248 |
| 0.586 | 0.246 | 1.000 | 0.504 | 1.000 | 0.119 | 0.136 | 0.102 | 0.207 | 0.523 | 0.116 | 0.258 |
| 0.652 | 0.251 | 1.000 | 0.485 | 1.000 | 0.118 | 0.145 | 0.111 | 0.207 | 0.595 | 0.122 | 0.245 |
| 0.556 | 0.257 | 0.930 | 0.448 | 0.900 | 0.121 | 0.148 | 0.106 | 0.193 | 0.545 | 0.121 | 0.258 |
| 0.634 | 0.270 | 0.900 | 0.434 | 0.920 | 0.120 | 0.149 | 0.114 | 0.209 | 0.523 | 0.111 | 0.248 |
| 0.640 | 0.286 | 1.000 | 0.462 | 1.000 | 0.102 | 0.142 | 0.106 | 0.179 | 0.550 | 0.122 | 0.269 |
| 0.716 | 0.270 | 0.900 | 0.439 | 0.920 | 0.111 | 0.149 | 0.108 | 0.201 | 0.556 | 0.133 | 0.261 |
| 0.550 | 0.281 | 0.910 | 0.448 | 0.920 | 0.124 | 0.151 | 0.098 | 0.187 | 0.595 | 0.114 | 0.256 |
| 0.592 | 0.267 | 0.910 | 0.480 | 0.920 | 0.102 | 0.149 | 0.117 | 0.219 | 0.550 | 0.117 | 0.248 |
| 0.622 | 0.243 | 0.910 | 0.444 | 0.920 | 0.104 | 0.144 | 0.104 | 0.187 | 0.534 | 0.129 | 0.277 |
| 0.598 | 0.270 | 0.910 | 0.476 | 0.920 | 0.115 | 0.157 | 0.098 | 0.213 | 0.589 | 0.114 | 0.258 |
| 0.640 | 0.273 | 0.930 | 0.420 | 0.920 | 0.120 | 0.145 | 0.103 | 0.179 | 0.550 | 0.512 | 0.253 |
| 0.664 | 0.270 | 0.940 | 0.453 | 0.920 | 0.106 | 0.148 | 0.109 | 0.183 | 0.606 | 0.123 | 0.261 |
| 0.550 | 0.289 | 0.950 | 0.420 | 0.920 | 0.124 | 0.158 | 0.111 | 0.215 | 0.500 | 0.135 | 0.253 |
| 0.604 | 0.254 | 0.960 | 0.485 | 0.920 | 0.104 | 0.155 | 0.098 | 0.193 | 0.517 | 0.127 | 0.272 |
| 0.604 | 0.297 | 0.930 | 0.453 | 0.900 | 0.107 | 0.163 | 0.101 | 0.193 | 0.589 | 0.130 | 0.272 |
| 0.634 | 0.270 | 0.980 | 0.420 | 0.920 | 0.118 | 0.145 | 0.110 | 0.219 | 0.584 | 0.132 | 0.275 |
| 0.616 | 0.275 | 0.990 | 0.494 | 0.920 | 0.115 | 0.139 | 0.113 | 0.211 | 0.612 | 0.133 | 0.267 |
| 0.658 | 0.284 | 0.990 | 0.480 | 0.920 | 0.112 | 0.160 | 0.103 | 0.217 | 0.573 | 0.125 | 0.269 |
| 0.646 | 0.248 | 1.000 | 0.425 | 0.920 | 0.103 | 0.142 | 0.114 | 0.207 | 0.539 | 0.125 | 0.261 |
| 0.580 | 0.257 | 1.000 | 0.448 | 0.920 | 0.102 | 0.148 | 0.114 | 0.189 | 0.534 | 0.133 | 0.296 |
| 0.616 | 0.246 | 1.000 | 0.480 | 0.920 | 0.114 | 0.133 | 0.112 | 0.209 | 0.556 | 0.116 | 0.267 |
| 0.646 | 0.257 | 1.000 | 0.453 | 0.920 | 0.122 | 0.133 | 0.109 | 0.205 | 0.517 | 0.114 | 0.245 |
| 0.598 | 0.278 | 1.000 | 0.448 | 0.920 | 0.115 | 0.160 | 0.100 | 0.183 | 0.528 | 0.513 | 0.250 |
| 0.556 | 0.259 | 1.000 | 0.467 | 0.920 | 0.124 | 0.139 | 0.117 | 0.203 | 0.589 | 0.123 | 0.291 |
| 0.580 | 0.278 | 1.000 | 0.425 | 0.920 | 0.113 | 0.163 | 0.096 | 0.197 | 0.500 | 0.128 | 0.258 |
| 0.556 | 0.267 | 0.970 | 0.439 | 0.900 | 0.107 | 0.148 | 0.113 | 0.193 | 0.562 | 0.412 | 0.294 |
| 0.610 | 0.297 | 1.000 | 0.453 | 0.920 | 0.118 | 0.135 | 0.114 | 0.209 | 0.584 | 0.122 | 0.267 |
| 0.634 | 0.278 | 1.000 | 0.504 | 0.920 | 0.121 | 0.157 | 0.101 | 0.197 | 0.606 | 0.121 | 0.253 |
| 0.580 | 0.262 | 1.000 | 0.453 | 0.920 | 0.120 | 0.138 | 0.096 | 0.215 | 0.595 | 0.124 | 0.277 |
| 0.586 | 0.297 | 0.900 | 0.494 | 0.930 | 0.118 | 0.148 | 0.110 | 0.209 | 0.584 | 0.128 | 0.256 |
| 0.610 | 0.267 | 0.910 | 0.462 | 0.930 | 0.122 | 0.147 | 0.114 | 0.191 | 0.539 | 0.114 | 0.286 |
| 0.610 | 0.281 | 0.910 | 0.425 | 0.930 | 0.105 | 0.152 | 0.105 | 0.187 | 0.573 | 0.113 | 0.245 |
| 0.598 | 0.286 | 0.910 | 0.476 | 0.930 | 0.116 | 0.161 | 0.116 | 0.201 | 0.589 | 0.112 | 0.296 |
| 0.610 | 0.254 | 0.920 | 0.444 | 0.930 | 0.115 | 0.161 | 0.103 | 0.207 | 0.584 | 0.127 | 0.286 |
| 0.586 | 0.275 | 0.920 | 0.416 | 0.930 | 0.120 | 0.161 | 0.118 | 0.193 | 0.556 | 0.130 | 0.269 |
| 0.622 | 0.284 | 0.940 | 0.457 | 0.930 | 0.107 | 0.151 | 0.103 | 0.215 | 0.500 | 0.127 | 0.261 |
| 0.562 | 0.289 | 0.980 | 0.425 | 0.900 | 0.113 | 0.141 | 0.109 | 0.207 | 0.600 | 0.411 | 0.253 |
| 0.568 | 0.297 | 0.950 | 0.494 | 0.930 | 0.112 | 0.152 | 0.114 | 0.183 | 0.606 | 0.119 | 0.264 |
| 0.592 | 0.270 | 0.950 | 0.499 | 0.930 | 0.113 | 0.161 | 0.112 | 0.191 | 0.534 | 0.114 | 0.269 |
| 0.610 | 0.281 | 0.960 | 0.508 | 0.930 | 0.110 | 0.160 | 0.096 | 0.201 | 0.589 | 0.125 | 0.267 |
| 0.592 | 0.297 | 0.970 | 0.457 | 0.930 | 0.118 | 0.147 | 0.113 | 0.219 | 0.528 | 0.128 | 0.256 |
| 0.634 | 0.297 | 0.980 | 0.444 | 0.930 | 0.112 | 0.157 | 0.097 | 0.209 | 0.517 | 0.134 | 0.245 |
| 0.568 | 0.257 | 0.980 | 0.499 | 0.930 | 0.111 | 0.152 | 0.117 | 0.211 | 0.528 | 0.122 | 0.269 |
| 0.622 | 0.259 | 0.990 | 0.444 | 0.930 | 0.115 | 0.147 | 0.101 | 0.195 | 0.589 | 0.111 | 0.248 |
| 0.652 | 0.292 | 1.000 | 0.439 | 0.930 | 0.103 | 0.145 | 0.104 | 0.201 | 0.578 | 0.119 | 0.253 |
| 0.658 | 0.267 | 1.000 | 0.504 | 0.930 | 0.105 | 0.141 | 0.118 | 0.205 | 0.500 | 0.127 | 0.296 |
| 0.664 | 0.275 | 1.000 | 0.439 | 0.930 | 0.103 | 0.161 | 0.109 | 0.219 | 0.512 | 0.118 | 0.296 |
| 0.586 | 0.270 | 0.990 | 0.416 | 0.900 | 0.102 | 0.152 | 0.617 | 0.183 | 0.545 | 0.113 | 0.250 |
| 0.598 | 0.248 | 1.000 | 0.420 | 0.930 | 0.123 | 0.136 | 0.104 | 0.183 | 0.539 | 0.124 | 0.264 |
| 0.658 | 0.254 | 1.000 | 0.480 | 0.930 | 0.118 | 0.154 | 0.118 | 0.215 | 0.506 | 0.119 | 0.261 |
| 0.628 | 0.292 | 1.000 | 0.444 | 0.930 | 0.111 | 0.149 | 0.113 | 0.211 | 0.612 | 0.116 | 0.253 |
| 0.652 | 0.284 | 1.000 | 0.490 | 0.930 | 0.121 | 0.145 | 0.111 | 0.213 | 0.578 | 0.124 | 0.286 |
| 0.604 | 0.248 | 1.000 | 0.499 | 0.930 | 0.114 | 0.135 | 0.110 | 0.195 | 0.556 | 0.118 | 0.245 |
| 0.616 | 0.265 | 1.000 | 0.480 | 0.930 | 0.102 | 0.151 | 0.102 | 0.215 | 0.567 | 0.119 | 0.250 |
| 0.640 | 0.286 | 1.000 | 0.467 | 0.930 | 0.103 | 0.161 | 0.096 | 0.207 | 0.517 | 0.116 | 0.256 |
| 0.562 | 0.243 | 1.000 | 0.434 | 0.930 | 0.113 | 0.144 | 0.097 | 0.193 | 0.523 | 0.123 | 0.296 |
| 0.622 | 0.278 | 1.000 | 0.448 | 0.930 | 0.105 | 0.157 | 0.108 | 0.189 | 0.523 | 0.132 | 0.288 |
| 0.610 | 0.270 | 0.910 | 0.453 | 0.940 | 0.123 | 0.136 | 0.102 | 0.203 | 0.578 | 0.124 | 0.261 |
