# Supplementary material for: An assessment model of bio-efficiency for container terminals in the presence of air emissions
Source: PLoS One. 2025 Mar 19;20(3):e0319423. doi: 10.1371/journal.pone.0319423 (PMC11957765; doi:10.1371/journal.pone.0319423)
Supplement: Attachment — Submitted filename: Comments.docx [file pone.0319423.s002.docx]

Dear authors,

This paper aims to assess bio-efficiency for container terminals (CTs) in the presence of air emissions utilizing a slacks-based measure (SBM) model. I can say that assessing bio-efficiency in container terminals is a timely and significant topic, especially given the global push for sustainability and reducing carbon footprints. The focus on air emissions aligns with the growing environmental concerns in logistics and port management. Nonetheless, this paper will be better for Plos One if some comments below are addressed in the revised manuscript:

1. Explain why cluster analysis is necessary in this paper.
2. Ensure that CO2 emissions are clearly determined. For example, if your focus is on measuring bio-efficiency, explicitly state what metrics you are using to calculate CO2 emissions at CTs.
3. Strengthen the link between your study's objectives and the practical implications for container terminal operations. Emphasizing how your findings can inform policymakers or terminal operators will increase the paper's impact.
4. Your literature review should cover the latest studies on bio-efficiency, port emissions, and sustainable logistics. Consider including references to recent developments in green port initiatives, such as the use of shore power or electric cranes.
5. In this paper, you used Data Envelopment Analysis to determine bio-efficiency. Explain why it's best suited for analyzing bio-efficiency in container terminals.
6. In your conclusion, reiterate the main contributions of your study and how it advances the field. This is especially important if you're proposing new methods or frameworks for assessing bio-efficiency.
7. Minor revisions: This paper should be checked English grammars/errors by an English native speaker.

Best regards,
