# Supplementary material for: An assessment model of bio-efficiency for container terminals in the presence of air emissions
Source: PLoS One. 2025 Mar 19;20(3):e0319423. doi: 10.1371/journal.pone.0319423 (PMC11957765; doi:10.1371/journal.pone.0319423)
Supplement: Attachment — Submitted filename: R2. Responses.Lan.docx [file pone.0319423.s004.docx]

Dear Editor-in-chief and Reviewers,

We sincerely thank you for the time and effort you have dedicated to reviewing our manuscript titled “An assessment model of bio-efficiency for container terminals in the presence of air emissions (manuscript ID: PONE-D-24-41055)." We greatly appreciate your valuable feedback and constructive suggestions, which have significantly contributed to improving the quality and clarity of our work.

In response to your comments, we have carefully revised the manuscript and addressed all points raised. Below, we provide a detailed summary of the changes made and our responses to each of the reviewers' comments. We believe these revisions have enhanced the manuscript, and we hope that the revised version meets the standards of PLOS ONE.

Thank you once again for your thoughtful input. We look forward to your continued feedback.

Sincerely,

| **Comments** | **Responses** | **Location in Manuscript** |
| --- | --- | --- |
| Comment # 1. Review grammar and writing style. | **Response**: Thanks so much for your comments. We have our manuscript proofread by a native English speaker to ensure it is free of grammatical errors and writing style issues. | The revisions have reflected across our manuscript. |
| Comment # 2. Check mathematical symbols, especially verify and further explain formulas (3), (4), (5) (lines 260 to 262). | **Response**: Thanks so much for your comments. We checked these formulas and corrected where necessary. | These revisions have been incorporated in Rows 260-268. |
| Comment # 3. Expand the conclusion to highlight the contributions of the paper. The section on research limitations should be separated into a smaller section (e.g., 5.2) | **Response**: Thanks so much for your comments. We revised our manuscript. Particularly, the conclusions section was separated into two subsections: (1) “5.1. Conclusions” and “5.2. Research limitations”. | These changes are reflected in Rows 369-423. |
